# Peripapillary Intrachoroidal Cavitation in High Myopia: A Case Report

**DOI:** 10.7759/cureus.105212

**Published:** 2026-03-14

**Authors:** Rania Lakraa, Marouane Kanboui, El Hassan Abdallah

**Affiliations:** 1 Department of Ophthalmology B, Ibn Sina University Hospital, Rabat, MAR

**Keywords:** high myopia, optical coherence tomography, pathologic myopia, peripapillary intrachoroidal cavitation, posterior staphyloma

## Abstract

Peripapillary intrachoroidal cavitation (PICC) is an increasingly recognized structural alteration predominantly observed in highly myopic eyes and considered part of the spectrum of myopic posterior segment remodeling. Progressive axial elongation and associated biomechanical stress at the level of the optic nerve head and peripapillary region are thought to contribute to localized choroidal splitting and cavitation formation. The advent of enhanced depth imaging and swept-source optical coherence tomography (SS-OCT) has significantly improved the visualization of the choroid, allowing the precise characterization of PICC as a well-demarcated hyporeflective intrachoroidal space typically located inferior to the optic disc, beneath an intact retinal pigment epithelium-Bruch’s membrane complex.

We report the case of a highly myopic patient in whom swept-source OCT revealed a peripapillary intrachoroidal cavitation associated with localized choroidal thinning and peripapillary atrophy, without evidence of choroidal neovascularization or subretinal fluid. The diagnosis was established based on multimodal imaging findings. The main differential diagnoses, including pigment epithelial detachment, peripapillary staphyloma, and glaucomatous excavation, were carefully excluded.

This case highlights the importance of recognizing the characteristic OCT features of PICC to avoid misdiagnosis and unnecessary therapeutic interventions. A better understanding of its imaging profile and clinical associations is essential, particularly in highly myopic patients in whom structural optic nerve head changes may complicate interpretation. Periodic structural and functional monitoring is recommended given the potential association with visual field abnormalities.

## Introduction

High myopia is associated with progressive posterior segment remodeling, including choroidal thinning and optic nerve head deformation. According to Ikuno, high myopia encompasses a wide spectrum of structural complications affecting the posterior pole [1]. Similarly, Ng et al. highlighted the role of advanced optical coherence tomography (OCT) imaging in characterizing myopic structural changes [2].

Peripapillary intrachoroidal cavitation (PICC) is characterized by a hyporeflective choroidal space adjacent to the optic disc, best visualized using OCT. This entity was reappraised and structurally defined by Wei et al. using OCT imaging [3]. Epidemiological data from the Beijing Eye Study by You et al. [4] and from the Zhongshan High Myopia Cohort reported by Liu et al. [5] indicate a prevalence of approximately 3%-4% among highly myopic eyes. The recognition of PICC is essential to avoid confusion with pigment epithelial detachment or glaucomatous changes, as emphasized by Faghihi et al. [6] and more recently by Stephenson et al. [7].

## Case presentation

A 58-year-old woman with a long-standing high myopia presented with mild visual distortion in her left eye. She denied acute visual loss, photopsia, or floaters. Her refractive error was -15.00 diopters bilaterally, and she had no history of ocular surgery, trauma, or glaucoma.

Best-corrected visual acuity was 20/25 in the right eye and 20/30 in the left eye. Intraocular pressure was within normal limits in both eyes. Anterior segment examination was unremarkable. Fundus examination revealed a tilted optic disc with a large temporal myopic conus and marked parapapillary atrophy.

**Figure 1:**
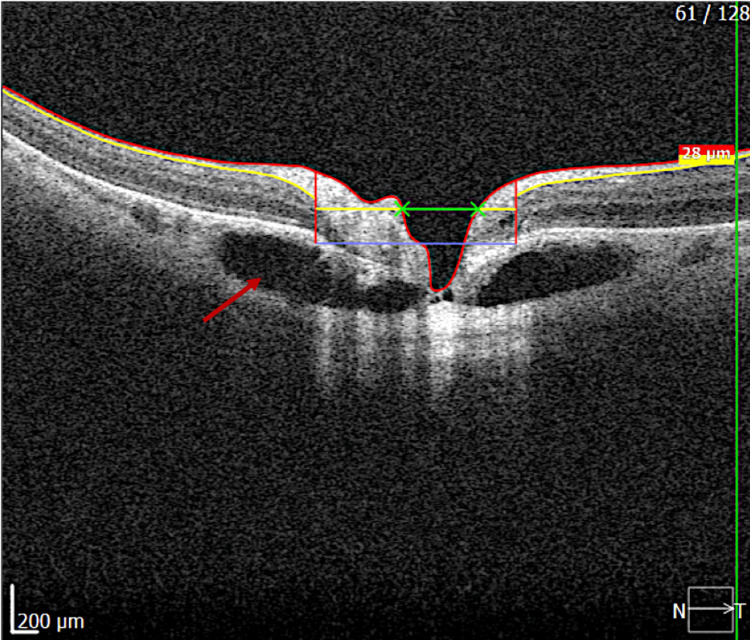
Swept-source optical coherence tomography (OCT) of the peripapillary region. A well-demarcated hyporeflective intrachoroidal cavity is visible inferior to the optic nerve head, associated with localized choroidal thinning (red arrow). The retinal pigment epithelium and Bruch’s membrane complex remain intact.

**Figure 2:**
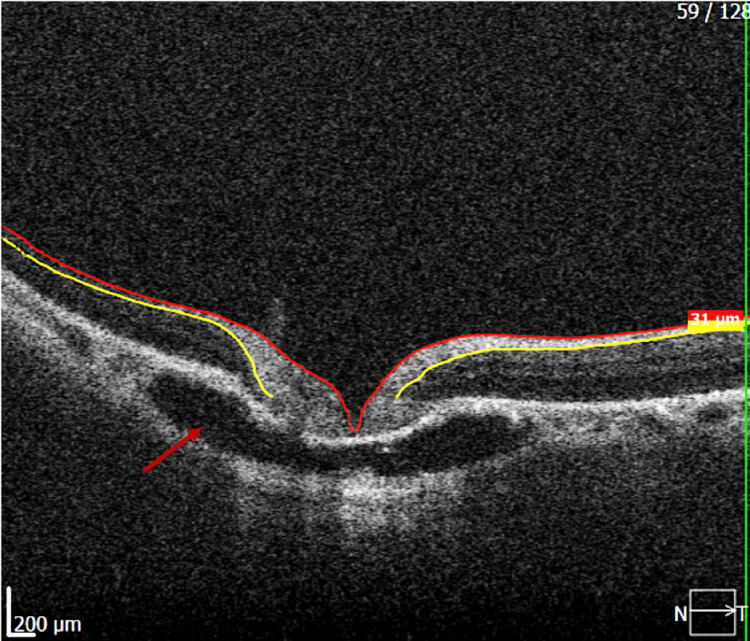
This superior B-scan at the level of the optic nerve head demonstrates the confluence of the intrachoroidal cavities on either side of the optic disc excavation, forming a continuous cavitary space (red arrow).

Swept-source optical coherence tomography (SS-OCT) of the peripapillary region revealed a well-defined hyporeflective intrachoroidal cavity located inferior to the optic nerve head, associated with localized choroidal thinning, while the retinal pigment epithelium-Bruch’s membrane complex remained intact (Figure 1). A superior B-scan at the level of the optic nerve head demonstrated the confluence of the intrachoroidal cavities on either side of the optic disc excavation, forming a continuous cavitary space (Figure 2). No subretinal fluid or signs of choroidal neovascularization were detected. These findings were consistent with a diagnosis of peripapillary intrachoroidal cavitation.

Given the absence of associated complications, no treatment was initiated. The patient was managed conservatively with periodic clinical and OCT monitoring. During follow-up, visual acuity remained stable, and no progression of the cavitation or secondary complications was observed.

## Discussion

Peripapillary intrachoroidal cavitation (PICC) is recognized as a structural manifestation within the spectrum of pathologic high myopia. As described by Ikuno, posterior pole remodeling in high myopia results from progressive axial elongation [1]. Advanced OCT imaging techniques, as detailed by Ng et al., have significantly improved the understanding of these structural changes [2].

The intrachoroidal nature of PICC was clearly demonstrated by Wei et al., who showed that the lesion corresponds to a hyporeflective space confined within the choroid [3]. Structural OCT findings in highly myopic eyes were further detailed by Faghihi et al., supporting the concept of localized choroidal remodeling [6]. Large cohort analyses by You et al. [4] and Liu et al. [5] confirmed that PICC is not uncommon in highly myopic populations.

Functionally, visual field alterations have been reported by Chen et al., suggesting a potential association with deep optic nerve head structural changes [8]. Additionally, OCT angiography findings described by Akiyama et al. demonstrated reduced peripapillary vessel density in affected eyes, although the clinical implications of these vascular alterations remain uncertain [9].

Recent observations by Stephenson et al. [7] and Ehongo et al. [10] further emphasized the importance of distinguishing PICC from other peripapillary myopic changes. The differential diagnosis includes other peripapillary changes observed in highly myopic eyes, such as peripapillary staphyloma, myopic peripapillary atrophy, or glaucomatous excavation. In this case, the presence of a well-defined hyporeflective intrachoroidal space on OCT, with the preservation of the retinal pigment epithelium-Bruch’s membrane complex and the absence of features suggestive of alternative entities, supports the diagnosis of peripapillary intrachoroidal cavitation. The recognition of the characteristic OCT features is essential to avoid misdiagnosis and unnecessary treatment, particularly in patients with concomitant optic nerve head abnormalities.

## Conclusions

Peripapillary intrachoroidal cavitation is an underrecognized structural alteration associated with pathologic high myopia that can be accurately identified using modern optical coherence tomography. The recognition of its characteristic imaging features is essential to distinguish it from other peripapillary abnormalities, including pigment epithelial detachment and glaucomatous excavation. This distinction is particularly important to prevent diagnostic confusion and unnecessary therapeutic interventions. Careful multimodal imaging evaluation and regular follow-up remain recommended in highly myopic patients presenting with peripapillary structural changes.
